# Synthesis and Study of Properties of Waterborne Polyurethanes Based on β-Cyclodextrin Partial Nitrate as Potential Systems for Delivery of Bioactive Compounds

**DOI:** 10.3390/polym14235262

**Published:** 2022-12-02

**Authors:** Sergei V. Karpov, Aigul S. Dzhalmukhanova, Vladimir G. Kurbatov, Eugenia O. Perepelitsina, Alexander E. Tarasov, Elmira R. Badamshina

**Affiliations:** 1 Department of Polymers and Composite Materials, Federal Research Center of Problems of Chemical Physics and Medicinal Chemistry, Russian Academy of Sciences, Academician Semenov Avenue 1, Chernogolovka 142432, Russia; 2 Department of Chemical Technology of Organic Coatings, Yaroslavl State Technical University, Moscow Avenue 88, Yaroslavl 150023, Russia

**Keywords:** waterborne polyurethane, β-cyclodextrin partial nitrate, hydrodynamic parameters, rheological behavior, thermal properties

## Abstract

Eco-friendly waterborne polyurethanes (WPU) find wide application in agriculture as pesticide carriers, which enhances their efficiency. To provide better control of the retention time and capacity of pesticides, WPU can be modified by cyclodextrin derivatives able to form supramolecular assemblies with bioactive substances. Synthesis of WPU containing up to 15 wt.% of covalently bound β-cyclodextrin partial nitrate (CDPN) is reported in this work. Covalent bonding of CDPN to a polyurethane matrix has been proved by IR spectroscopy and size exclusion chromatography. The particle size and viscosity of the WPU dispersion have been determined. The introduction of CDPN affects molecular weight and thermal properties of WPU films. The presence of CDPN in WPU is shown to provide higher average molecular weight, wider molecular weight distribution, and larger average size of dispersed particles, compared with WPU reference samples containing 1,4-butanediol. The analysis of the rheological behavior of the obtained WPU dispersions shows that they can be classified as pseudoplastic liquids. The analysis of the thermal parameters of WPU films indicates that the introduction of 15.0 wt.% CDPN shifts the value of the glass transition temperature from −63 °C to −48 °C compared with reference samples. We believe that the results of the present study are sufficiently encouraging in terms of using CDPN-modified eco-friendly WPU as potential systems for developing the delivering agents of bioactive compounds. The application of such systems will allow the long-term contact of pesticides with the plant surface and minimize the possibility of their release into the environment.

## 1. Introduction

Synthesis of eco-friendly waterborne polyurethanes (WPU) is one of most intensively developed fields of polymer chemistry [1,2,3,4,5]. WPU are amphiphilic polymers, the hydrophilic parts of which are considered to be urethane fragments bound with ionic centers, and hydrophobic parts are urethane fragments of oligo ethers and low-molecular-weight diols/diamines. Thus, WPU tend to self-organize and can form micelles or vesicles [6,7,8]. Due to their amphiphilic nature, WPU are used as pesticide delivery systems, which provides their controlled release [9,10,11,12].

The higher efficiency of pesticides is achieved by the encapsulation of bioactive compounds in a nonpolar WPU core. The so-prepared nanoemulsions deposited on plant leaves provide the presence of pesticides in contact with potential plant pests for a long period of time. The efficiency of the retention of pesticides encapsulated in WPU is noticeably higher than that of commercial compositions available as concentrates or powders due to the low surface tension of these emulsions and the possibility of forming hydrogen bonds between the leaf surface and a polymer matrix [11].

It is obvious that WPU loading capacity relative to pesticides and ability to control retention time are determined by both the nature of a bioactive compound and the components of a polymer matrix. To improve these characteristics, WPU can be modified by compounds able to form supramolecular assemblies with pesticides. It is known that cyclodextrins, which are macrocyclic compounds containing six, seven, or eight glucose fragments (α, β, and γ cyclodextrins) are able to form host–guest-type inclusion complexes with a wide range of pesticides including insecticides, fungicides, herbicides, repellents, and growth hormones [13,14,15]. Depending on the number of glucose fragments involved in a macrocycle, cyclodextrins contain 18 (α-cyclodextrin), 21 (β-cyclodextrin), or 24 (γ-cyclodextrin) hydroxyl groups per molecule. Apparently, the use of such multi-functional compounds in the synthesis of WPU shall result in the formation of cross-linked products unable to form stable aqueous dispersions. Thus, we propose using β-cyclodextrin partial nitrates (CDPN) containing four hydroxyl groups per macrocycle on average [16] to obtain WPU. It can be suggested that, due to the presence of nitro groups, the CDPN can be nitrogen donors for agricultural crops as well.

To synthesize a polyurethane matrix as an isocyanate containing component, it is reasonable to use isophorone diisocyanate (IPDI). The choice of IPDI is stipulated by high hydrolysis resistance and the possibility of controlling urethane formation reactions due to different reactivity of isocyanate groups [17,18]. To enhance environmental safety, it is recommended to use biodegradable polycaprolactone diol (PCL) as oligo diol [8], and 2,2-bis(hydroxymethyl)propionic acid (DMPA) should be used to provide the hydrophilicity of a polyurethane matrix and the possibility of preparing stable aqueous dispersions [19].

It is expected that the use of pesticide delivery systems based on eco-friendly WPU modified by CDPN provides the possibility of a long-term contact between bioactive compounds and plant surface and minimizes the risk of their occasional release into the environment. As a result, it could lead to a lower anthropogenic impact on the environment and increase the efficiency of pesticide exploitation in agriculture.

This work is aimed at the synthesis and study of the properties of WPU containing covalently bound CDPN, which are promising systems for the delivery of bioactive compounds.

## 2. Materials and Methods

### 2.1. Materials

PCL (M_n_ = 2000 g/mol, Sigma-Aldrich, Geel, Belgium) and DMPA (Sigma-Aldrich, Geel, Belgium) were dried under vacuum for 12 h at 60 °C prior to use. IPDI (Sigma-Aldrich, Geel, Belgium) was purified by vacuum distillation at T = 90 °C and P = 13 mbar. Dibutyltin dilaurate (DBTDL) (Sigma Aldrich, Geel, Belgium), 1,4-butanediol (BD) (Sigma-Aldrich, Steinheim, Germany), 1,3-diaminopropane (DAP) (Sigma-Aldrich, Geel, Belgium), and triethylamine (TEA) (Sigma-Aldrich, Geel, Belgium) were used without further purification. Acetone was dried over molecular sieves 4A.

CDPN was synthesized at the Laboratory of Energetic Polymer Systems of Federal Research Center of Problems of Chemical Physics and Medicinal Chemistry RAS [16]. The content of the OH groups of 3.5 ± 0.1 wt.% corresponds to 4 hydroxyl groups per macrocycle.

### 2.2. Characterization

To determine the solid content, WPU dispersions (1 g) were placed in a Petri dish, and the solvent was evaporated at T = 105 °C until no change in weight was observed.

The IR spectra of WPU films were recorded with a Bruker Alpha FTIR spectrometer in attenuated total reflection mode (ATR) (Bruker, Ettlingen, Germany). The spectra were recorded in the 7000 ÷ 350 cm^−1^ range at 2 cm^−1^ resolution. The number of scans was 48.

The particle size distribution of WPU dispersions was recorded by a dynamic light scattering method at an angle of 160° at T = 25 °C using a Photocor Compact device (Photocor Instruments Inc., College Park, MD, USA).

The zeta potential of WPU dispersions was measured on a Zeta-sizer Nano ZS (Malvern Instruments, Malvern City, UK), and the samples were diluted to about 0.1 wt.% with distilled water before testing.

The rheological properties of WPU dispersions were analyzed with a Physica MCR-702 (Anton Paar, Graz, Austria) rheometer. Tests were carried out in a rotational mode at a controlled shear rate in the 1 to 100 s^–1^ range, as well as in an oscillatory mode, which corresponded to the range of the linear viscoelastic behavior of the tested systems (0.1–600 s^−1^ frequency range; deformation amplitude was 0.01%). A plane-to-plane measuring system was used; the plane diameter was 50 mm, and the gap between the planes was 1 mm. The tests were carried out at T = 30 °C.

The thermal behavior of the WPU films was investigated using a METTLER TOLEDO DSC822e (METTLER TOLEDO, Henniez, Switzerland) differential scanning calorimeter. Two heating and cooling cycles were carried out in the temperature range between −100 and 150 °C, under an N_2_ atmosphere at a heating rate of 10 °C/min. The glass transition temperature (T_g_) was determined at the mid-point of the glass transition after the second scan.

Thermal stability of the WPU films in the 30 to 600 °C range was studied using thermogravimetric analysis (NETZSCH STA 449 F3 Jupiter, Selb, Germany) at a heating rate of 10 °C/min under an N_2_ atmosphere.

The molecular weight characteristics of WPU were determined by size exclusion chromatography (SEC) with a Waters GPCV 2000 chromatograph (PL-gel column, 5 μm, MIXED-C, 300 mm × 7.5 mm, Waters Corporation, Milford, MA, USA) equipped with a refractometric detector. N-methylpyrrolidone with 0.5 wt.% LiCl was used as eluent. The measurements were carried out at T = 70 °C; the elution rate was 1 mL/min. The experimental data were processed using the “Empower” and Astra program packages, version 5.3.2.20 (Wyatt Technology, Santa Barbara, CA, USA).

### 2.3. Preparation of WPU Dispersions and Films

**Figure 1:**
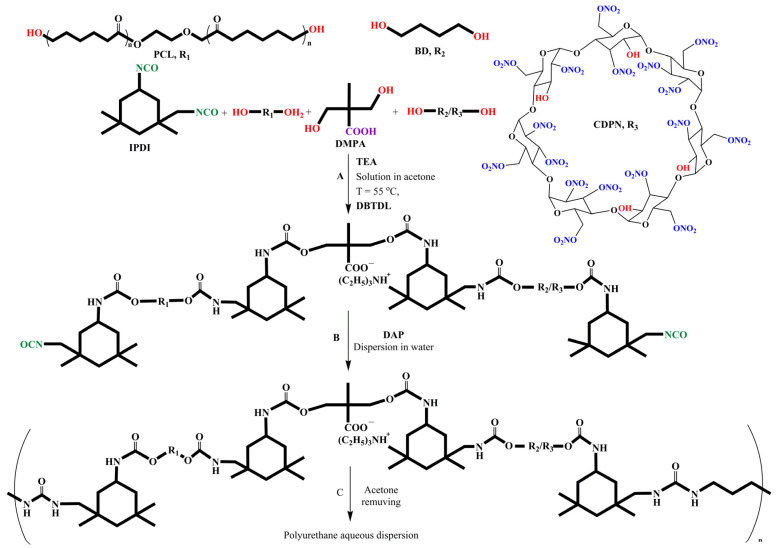
WPU synthesis scheme.

The synthetic scheme of WPU dispersions with CDPN or BD is shown in Figure 1. All samples of WPU dispersions were synthesized in acetone under an argon atmosphere. A [NCO]/[OH] ratio was 2.0 at the stage of pre-polymer synthesis. The concentration of a DBTDL catalyst was 0.05 mol/l. The ratio of starting reagents is listed in Table 1.

The WPU films were obtained by casting the prepared WPU dispersions, drying for 24 h at room temperature, and further drying at T = 60 °C under vacuum.

At the first step of the synthesis (A on Figure 1), DMPA, TEA, and acetone were placed in a flat-bottom flask with a ground neck and stirred with a magnetic stirrer at room temperature. When the reagents dissolved, PCL was added to the reaction mixture, which was stirred until the formation of a homogeneous mixture at T = 35 °C. The calculated amount of BD or CDPN was added. The reaction mass was cooled down to T = 25 °C, and IPDI and DBTDL catalyst were added to the reaction mixture. The moment of catalyst introduction was considered to be the start of the reaction. The urethane formation reaction proceeded upon stirring for 3 h at T = 55 °C. The conversion of isocyanate groups was determined by a standard di-n-butylamine titration method.

At the second step (B on Figure 1), the pre-polymer chain extension reaction was accompanied by dispersion. The dispersion was obtained by the addition of aqueous solution of DAP to the intensely stirred reaction mass. When the dispersion formed, acetone was removed by distillation under vacuum (C on Figure 1). The solid content was 18.2 ± 0.9 wt.% in all the samples.

The critical micelle concentration for all of the studied samples of aqueous polyurethane dispersions, determined by the light scattering method [20], was reached in the range of 1.1 to 1.8 wt.% of WPU content.

## 3. Results and Discussion

The samples of WPU dispersions were prepared in experiments contained 5.0, 10.0, and 15.0 wt.% of covalently bound CDPN. The content of a hydrophilizing agent was 5.0, 7.5, and 10.0 wt.% in each sample (samples 1.1–1.9, Table 2).

CDPN should be considered a fragment, which forms hard segments in polyurethanes. Therefore, to reveal the effect of CDPN covalently bound with a polyurethane matrix on molecular weight, hydrodynamic, rheological, thermophysical and thermochemical characteristics of WPU and their aqueous dispersions, reference samples were prepared, in which CDPN was replaced by BD (samples 2.1–2.9, Table 2). Sample 2.1 is an equivalent of sample 1.1, etc., since a [NCO]/[OH] ratio equals 2 for both series at the first step of synthesis, and the content of a crystallizing soft PCL segment remained almost unchanged in similar samples.

To determine the WPU composition and establish the fact of the covalent binding of CDPN with a polyurethane matrix, all of the samples were analyzed by IR spectroscopy.

### 3.1. Determination of Structure of WPU Films by IR Spectroscopy

**Figure 2:**
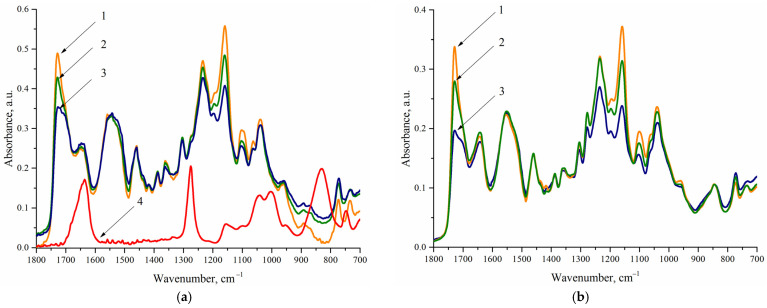
IR spectra for WPU films measured by ATR: (**a**) 1—sample 2.1; 2—sample 2.2; 3—sample 2.3; 4 –CDPN; (**b**) 1—sample 1.1; 2—sample 1.4; 3—sample 1.7.

IR spectra of CDPN and samples 2.1, 2.2, and 2.3 (Figure 2a, Table 3) were analyzed to reveal characteristic absorption bands for a hard macrocyclic fragment and a polyurethane matrix. Analysis of IR spectra presented in Figure 2a shows that, with equal absorption band intensities corresponding to the bending vibrations of the NH groups of urethane and urea fragments (~1540 cm^−1^) for samples 2.1–2.3, there are significant differences in the intensity of the absorption bands attributed to the valence vibrations of the carbonyl groups of PCL (~1727 cm^−1^). The revealed differences are in full agreement with the composition of WPU. In particular, Table 2 shows that the content of PCL is 50.8 wt.% in sample 2.1 and 39.5 wt.% in sample 2.3.

A comparative analysis of IR spectra for WPU samples 2.1–2.3 and CDPN shows that characteristic bands of antisymmetric (~1635 cm^−1^) and symmetric (~1275 cm^−1^) valence vibrations of nitrate groups are superimposed on those of polyurethanes. Thus, the band at 830 cm^−1^ attributed to the deformation vibrations of nitro groups should be used to identify CDPN in the composition of WPU.

**Figure 3:**
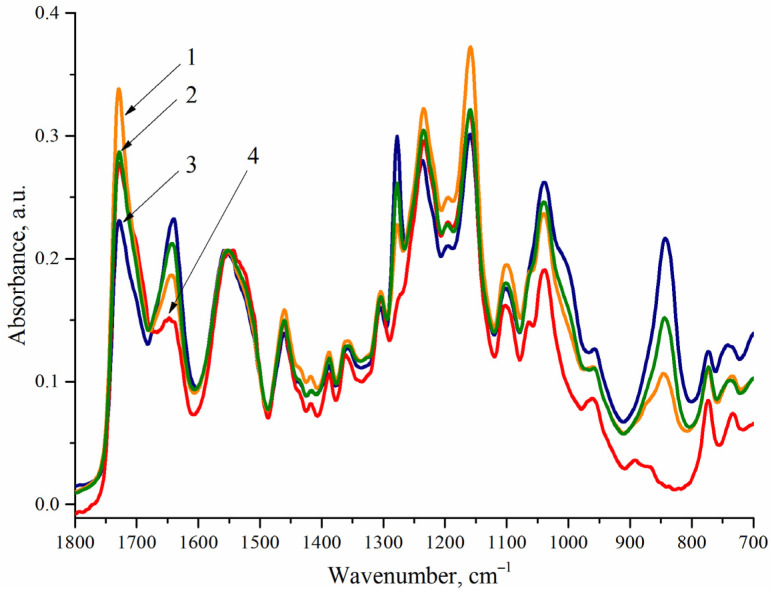
IR spectra for WPU films measured by ATR: 1—sample 1.1; 2—sample 1.2; 3—sample 1.3; 4—sample 2.2.

The IR spectra of samples 1.1, 1.4, and 1.7 (Figure 2b, Table 3) were analyzed to establish the covalent binding of CDPN with a polymer matrix of WPU. Since the CDPN content is the same in the studied samples (5.0 wt.%), the IR spectra are normalized to the band at 830 cm^−1^. It is seen that almost similar intensity of the characteristic bands of carbonyl fragments (~1645 cm^−1^), nitro groups (~1635 cm^−1^), and imino groups (~1540 cm^−1^) is observed for the discussed series of the samples. Therefore, the content of urethane, urea, and nitrate fragments is the same in all of the samples that corresponds to the composition of WPU presented in Table 2. Thus, it can be assumed that CDPN is covalently bound to a polyurethane matrix. The IR spectra of samples 1.1–1.3 and 2.2 normalized to the band at ~1540 cm^−1^ should be analyzed to confirm this assumption (Figure 3, Table 3).

It is seen from Figure 3 that, with an equal content of urethane and urea groups in the samples, the ratio of the intensity of the absorption bands of nitrate groups (~830 cm^−1^) for WPU samples 1.1–1.3 is close to 1:2:3, which corresponds to the content of CDPN in them (5.0, 10.0, and 15.0 wt.%, Table 2).

The given experimental data are an unambiguous proof, which can be obtained using IR spectroscopy, that CDPN is covalently bound to a polyurethane matrix, and the composition of WPU corresponds to the data listed in Table 2.

### 3.2. Investigations of the Molecular Weight Characteristics of WPU

All of the samples were analyzed by SEC. It should be noted that samples 1.1–1.9 lose solubility after the removal of the dispersion medium that can be stipulated by the formation of a physical 3D network of hydrogen bonds. In this regard, aqueous dispersions of WPU were used to analyze their molecular weight characteristics. 

**Figure 4:**
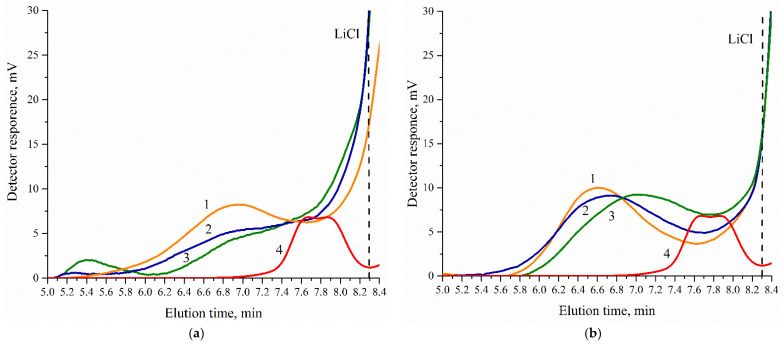
Chromatograms of WPU samples: (**a**) 1—sample 1.3; 2—sample 1.6; 3—sample 1.9; 4—CDPN; (**b**) 1—sample 2.3; 2—sample 2.6; 3—sample 2.9; 4—CDPN.

Figure 4a shows the chromatograms of individual CDPN and samples 1.3, 1.6, and 1.9, in which the CDPN content is 15.0 wt.%. To compare different samples and identify corresponding relationships, chromatograms were normalized in the 5.0–8.3 min elution time range. Moreover, the chromatogram of CDPN was normalized for its content in the WPU. The signal of CDPN is observed in the 7.0–8.3 min elution time range. It should be noted that no pronounced peak is observed in the chromatograms of samples 1.3, 1.6, and 1.9 in the same range. Since the signal intensity of a refractometric detector is different from zero, it is impossible to judge unambiguously the covalent binding of CDPN to a polymer chain. CDPN can remain in a free state, and its peak coincides with that of a low-molecular-weight fraction in a chromatogram.

The covalent binding of β-cyclodextrin nitro derivatives realized during the synthesis of WPU samples is confirmed by the chromatograms of CDPN free samples 2.3, 2.6, and 2.9 (Figure 4b). Signal intensity in the CDPN elution time range is different from zero as well, as described above.

Thus, taking into account the data of the IR analysis of the studied samples, we can state with a high degree of probability that CDPN is covalently bound to a polyurethane matrix.

The molecular weight characteristics of the WPU samples are listed in Table 4. It should be noted that the values of number average (M_n_) and weight average (M_w_) molecular weights are evaluative, since the signal of the low-molecular-weight fraction overlaps that of LiCl. However, it should be noted that it affects the value M_n_ to a greater extent.

The values of M_w_ of samples 2.1–2.9 tend to decrease due to the lower content of PCL in them. Attention should be paid to the fact that both M_w_ and M_w_/M_n_ are higher for samples 1.1–1.9 than for their CDPN-free equivalents. Most probably, this is due to the branched structure of the sample of series 1 formed as a result of the β-cyclodextrin derivatives used in experiments that contain four hydroxyl groups per macrocycle.

### 3.3. Analysis of Hydrodynamic Parameters and Zeta Potential of WPU Dispersions

Particle size was studied in aqueous polyurethane dispersions by dynamic light scattering. The data from processing the curves of particle size distribution of the dispersed phase of WPU are shown in Table 4.

**Figure 5:**
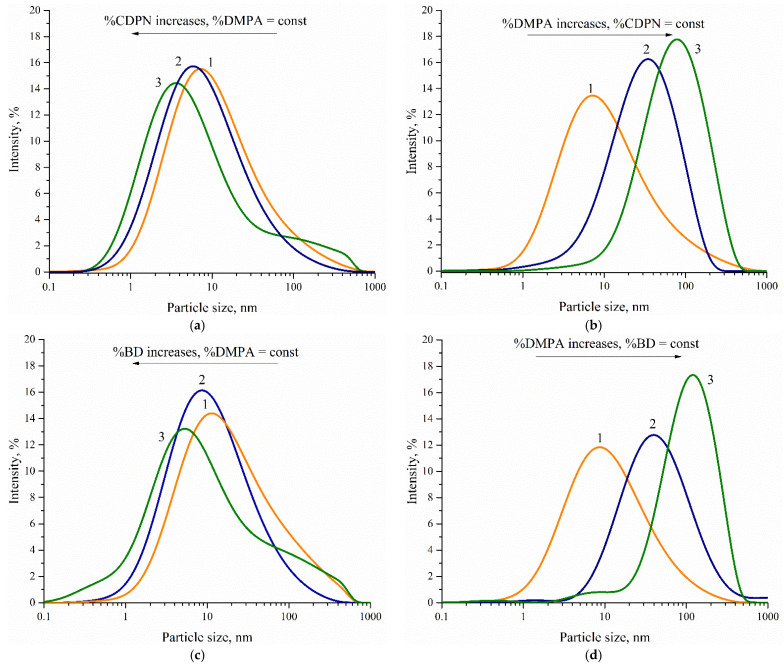
Typical curves of dispersed phase particle size distribution in WPU dispersion: (**a**) 1—sample 1.1, 2—sample 1.2, 3—sample 1.3; (**b**) 1—sample 1.1, 2—sample 1.4, 3—sample 1.7; (**c**) 1—sample 2.1, 2—sample 2.2, 3—sample 2.3; (**d**) 1—sample 1.1, 2—sample 1.4, 3—sample 1.7.

Series of samples 1.1–1.3, 1.4–1.6, and 1.7–1.9 should be considered when studying the effect of the mass fraction of CDPN on aqueous dispersion particle size. Figure 5a shows typical curves of the particle size distribution of the dispersed phase for samples 1.1–1.3. It is seen that the particle size value tends to decrease with increasing content of hard segments at a constant mass fraction of DMPA. However, the particle size of the dispersed phase of WPU increases along with the mass fraction of the hydrophilizing agent at a constant CDPN content (samples 1.1, 1.4, and 1.7; Table 4, Figure 5b). Similar dependencies of the particle size of the dispersed phase are observed for the series of WPU samples 2.1–2.9 (Table 4, Figure 5c,d).

Based on the results of the study of the aqueous dispersions of WPU 1.1–2.1, 1.2–2.2 etc., we can conclude that the replacement of BD by 5.0 wt.% of CDPN at a constant [NCO]/[OH] ratio at the first step of synthesis and constant mass fraction of PCL results in a smaller particle size. The particle size of the dispersed phase grows with the mass fraction of the hard macrocyclic CDPN fragment up to 15.0 wt.%.

Thus, the average size of the particles of WPU dispersions modified by CDPN can be changed by varying the content of DMPA and CDPN in their composition.

The zeta potential was also determined for all aqueous polyurethane dispersions, the value of which lies in the range from −42.3 to −48.3 mV (Table 4). This enables us to conclude that the studied WPU dispersions are quite stable.

### 3.4. Analysis of Rheological Characteristics of WPU Dispersions

Rheological characteristics are one of the crucial factors that determine such important properties of WPU dispersions as a way of applying the polymer material and its performance.

**Figure 6:**
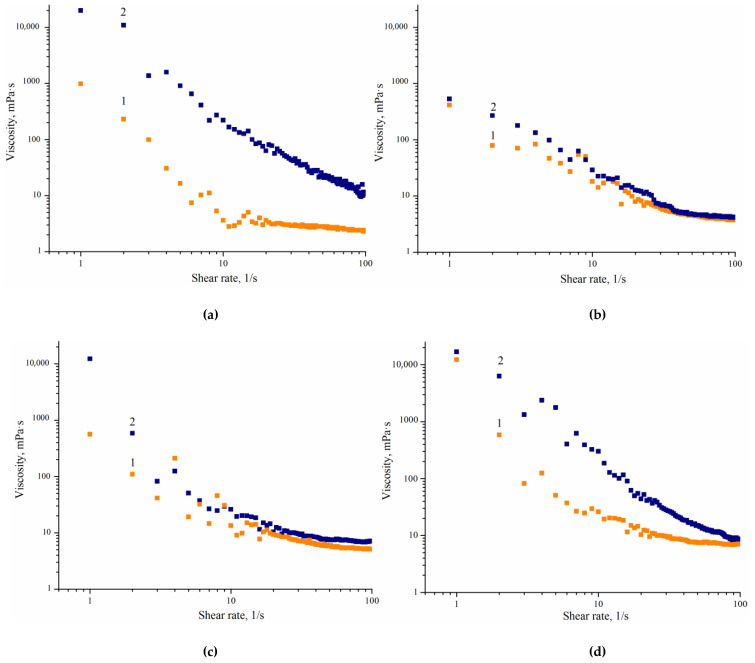
Viscosity of WPU dispersions as a function of shear rate: (**a**) 1—sample 1.1, 2—sample 2.1; (**b**) 1—sample 1.4, 2—sample 2.4; (**c**) 1—sample 1.7, 2—sample 2.7; (**d**) 1—sample 1.7, 2—sample 1.8.

Figure 6 shows typical viscosity curves for WPU dispersions. It is evident that all studied samples are characterized by a decrease in viscosity with an increasing shear rate. This is evidence that the studied systems can be classified as pseudoplastic liquids. The decrease in the dynamic viscosity of the system can be attributed to the destruction of an intermolecular interaction between particles with an increase in shear rate [30]. This can provide the high viscosity at zero shear required for their stability, combined with the low viscosity at a higher shear rate required for easy application with low energy consumption.

The aforementioned zero shear viscosity is one of the factors determining the stability of dispersions in storage. Since the products are stored in a state of low strain, they are affected by gravity force only. The lower the dispersion viscosity at a low shear rate, the higher the kinetic sedimentation that provides its stability, other things being equal [30]. The data presented in Figure 6 show that the expected value of zero shear viscosity is lower for the CDPN containing dispersions than for WPU prepared without using CDPN. Thus, it can be concluded that the stability of aqueous polyurethane dispersions containing β-cyclodextrin nitro derivatives is slightly lower in storage than that of BD-based WPU. It is also seen from Figure 6d that the increase in the content of CDPN from 5.0 to 10.0 wt.% has almost no effect on the stability of aqueous dispersions of WPU.

A series of tests was carried out using strain amplitude sweep to determine the range of the linear viscoelasticity of the studied water dispersions. It was found that the value of storage modulus G′ remains almost unchanged up to 0.01% of shear strain. Therefore, frequency sweep can be carried out at strains lower than 0.01% to provide a whole set of data in the range of linear viscoelasticity.

**Figure 7:**
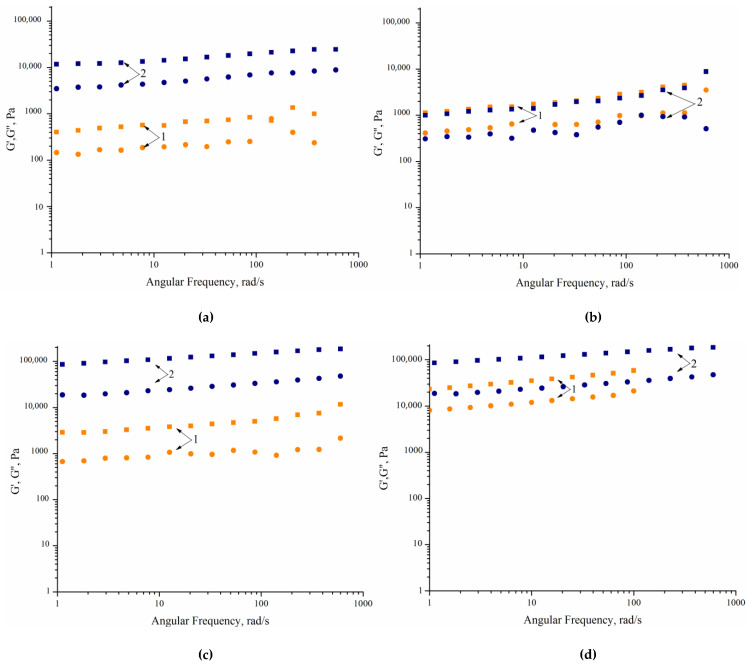
Frequency correlation of dynamic storage modulus G′ (solid squares) and loss modulus G″ (solid circles) for WPU dispersions (0.01% strain): (**a**) 1—sample 1.1, 2—sample 2.1; (**b**) 1—sample 1.4, 2—sample 2.4; (**c**) 1—sample 1.7, 2—sample 2.7; (**d**) 1—1.7, 2—sample 1.8.

Figure 7 shows the correlations between dynamic storage modulus G′ and loss modulus G″ on frequency determined from dynamic tests at a constant amplitude of 0.01% and frequency sweeping. The value of the dynamic storage modulus is higher than that of the loss modulus for all dispersions. The values of G′ and G″ are almost independent of frequency. Such behavior is typical for the formation of a fractal structure or gel and originates from a strong interaction between particles providing an optimal packing. The experimental evidence that G′ and G″ are frequency-independent is ascribed to the formation of equilibrium dynamic storage modulus G_eq_, which is a typical criterion for the formation of an elastic gel [31].

### 3.5. Analysis of the Thermophysical and Thermochemical Characteristics of WPU Films

The thermophysical and thermochemical characteristics of WPU films were studied by DSC and TGA. The experimental data are listed in Table 5. It is seen that the introduction of 15.0 wt.% of CDPN into the composition of WPU results in an increase in T_g_ by ~10 °C compared with the reference samples based on BD. Nevertheless, all of the T_g_ values are below −48 °C. Obviously, the high-elastic state of films in a broad temperature range will provide the release of bioactive compounds when WPU modified by CDPN is used as a pesticide delivery system.

**Figure 8:**
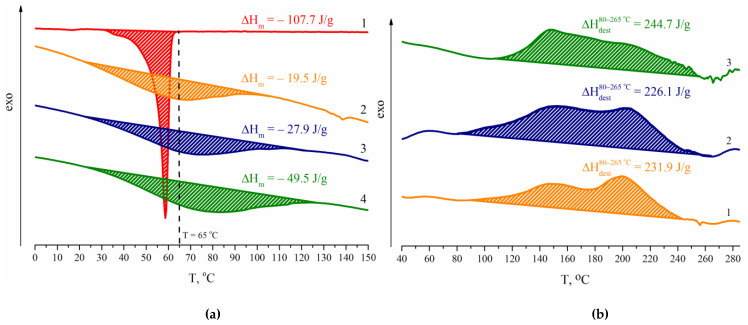
DSC thermograms for WPU films: (**a**) 1—PCL, 2—sample 2.1; 3—sample 2.4; 4—sample 2.7; (**b**) 1—sample 1.1; 2—sample 1.4; 3—sample 1.7.

Since PCL is a soft crystallizing segment, it should be expected that PCL-based WPU films tend to crystallize. Considering the studied samples to be close in chemical composition, the degree of their crystallinity can indirectly be evidenced from the value of enthalpy of the melting of a crystal (ΔH_m_) phase. Figure 8a shows DSC thermograms of WPU samples 2.1, 2.4, and 2.7 with a constant mass fraction of BD. The samples were prepared without using CDPN.

It seems reasonable that the degree of crystallinity decreases with the content of PCL in the studied series of the samples. However, the analysis of experimental values of the enthalpy of melting suggests the opposite. The observed experimental fact can be stipulated by different crystallization rates in the studied series of the samples. It should be noted that all of the samples melt in a wider temperature range compared with PCL. Seemingly, the crystal phase formed by hard segments melt at temperatures higher than 65 °C.

The DSC analysis of the CDPN containing WPU samples shows no endothermal peaks in the DSC thermograms (Figure 8b). It can be suggested that the presence of a β-cyclodextrin nitro derivative suppresses the crystallization of both soft and hard segments of a polyurethane matrix, in particular, due to its branched structure. It should be expected that the absence of a crystal phase will provide the release of bioactive compounds from the pesticide delivery systems based on WPU modified by CDPN.

The WPU–CDPN samples are characterized by heat release in the 80 to 265 °C range (ΔH_dest_^80−265 °C^), which is most likely due to the decomposition of nitro groups. ΔH_dest_^80−265 °C^ is ~230 J/g for WPU samples 1.1, 1.4, and 1.7 and is almost independent of DMPA content, as expected (Table 5). At the same time, the thermal effect of destruction increases with the increase in CDPN content in the series of samples 1.1–1.3, 1.4–1.6, and 1.7–1.9 (Table 5). Thus, the observed increase in the exothermal effect with CDPN content associated with the decomposition of nitro groups confirms the above assumption.

**Figure 9:**
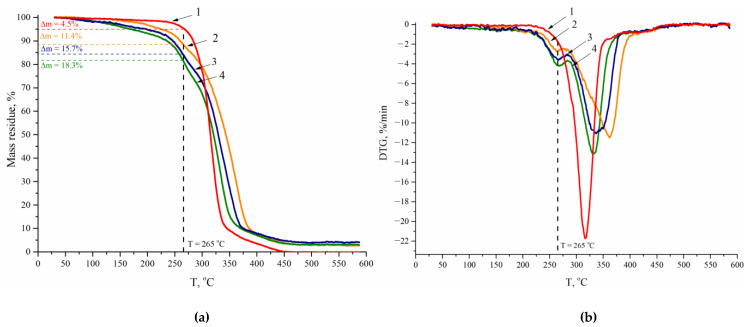
TGA (**a**) and DTGA (**b**) thermograms for WPU films: 1—sample 2.1; 2—sample 1.1; 3—sample 1.4; 4—sample 1.7.

The samples were also analyzed by TGA (Table 5, Figure 9a). Expectedly, the value of the loss of mass at temperatures corresponding to the end of the exothermal effect in the DSC thermograms (Δm_dest_^265 °C^, Table 5) of WPU samples 1.1–1.9 grows with the content of both CDPN and DMPA in their composition (see, for example, samples 1.1, 1.4, and 1.7).

The data presented in Figure 9b show that the thermal transformation of the WPU samples prepared without using CDPN is a one-step reaction. It is evidenced by the presence of one minimum on the differential TGA (DTGA) curves. It follows from the analysis of the thermal destruction of samples 1.1–1.9 containing β-cyclodextrin nitro derivatives that thermal transformation is a two-step reaction at the least. The first step can be assumed to be mainly the decomposition of nitro groups, while the second step is the destruction of a polymer matrix. The rate and degree of decomposition at the first step increase with DMPA content in the composition of WPU with constant CDPN content.

## 4. Conclusions

In the present work, the waterborne polyurethanes containing from 5 to 15 wt.% of covalently bound β-cyclodextrin partial nitrate have been synthesized. IR spectroscopy of WPU films proves that CDPN is covalently bound with a polymer matrix. These data are supported by SEC.

The properties of WPU containing hard CDPN and BD blocks, respectively, have been compared. The presence of CDPN provides a higher average molecular weight of WPU and a wider molecular mass distribution due to its branched structure. The formation of such a structure is stipulated by β-cyclodextrin derivatives being used in syntheses, which contain four hydroxyl groups per macrocycle.

The analysis of the hydrodynamic properties of WPU dispersions shows that the presence of 5.0 wt.% CDPN provides a smaller particle size compared with BD containing WPU, while particle size increases with CDPN content up to 15.0 wt.%. The particle size distribution is also affected by DMPA content in the WPU. Thus, the average size of particles of WPU dispersions modified by CDPN can be optimized to enhance their efficiency as pesticide delivery systems.

Rheological analysis of WPU dispersions is evidence that all studied samples can be classified as pseudoplastic liquids. It is shown that the introduction of CDPN into the WPU composition slightly lowers the kinetic sedimentation stability of the studied materials.

The analysis of the thermophysical and thermochemical characteristics of WPU films shows that T_g_ weakly depends on their composition and ranges from −63 °C to −48 °C. A noticeable increase in glass transition temperature by 15 °C is observed at 15.0 wt.% content of CDPN only. The replacement of BD by CDPN in a polymer matrix suppresses the crystallization of both soft and hard segments. The films of WPU modified by CDPN are in a highly elastic state in a wide temperature range. This fact, along with the absence of a crystal phase, will obviously provide the release of bioactive compounds from pesticide delivery systems based on WPU modified by CDPN.

Thus, it can be concluded that waterborne polyurethanes modified by β-cyclodextrin partial nitrate can be examined as bioactive compound delivery systems in agriculture.

## Figures and Tables

**Table 1:** Composition of WPU dispersions.

Sample	CDPN (OH), Mol	BD (OH), Mol	DMPA (OH), Mol	TEA, Mol	PCL (OH), Mol	IPDI (NCO), Mol	DAP (NH_2_), Mol
1.1	0.0010	0	0.0074	0.0037	0.0051	0.0270	0.0135
1.2	0.0020	0	0.0074	0.0037	0.0045	0.0278	0.0139
1.3	0.0030	0	0.0074	0.0037	0.0039	0.0286	0.0143
1.4	0.0010	0	0.0111	0.0056	0.0039	0.0320	0.016
1.5	0.0021	0	0.0111	0.0066	0.0034	0.0332	0.0166
1.6	0.0031	0	0.0111	0.0056	0.0028	0.0340	0.0170
1.7	0.0010	0	0.0148	0.0077	0.0029	0.0374	0.0187
1.8	0.0021	0	0.0148	0.0077	0.0023	0.0384	0.0192
1.9	0.0031	0	0.0148	0.0077	0.0017	0.0392	0.0196
2.1	0	0.0026	0.0074	0.0037	0.0051	0.0302	0.0151
2.2	0	0.0052	0.0074	0.0037	0.0045	0.0342	0.0171
2.3	0	0.0073	0.0074	0.0037	0.0040	0.0374	0.0187
2.4	0	0.0026	0.0111	0.0056	0.0039	0.0352	0.0176
2.5	0	0.0052	0.0111	0.0066	0.0033	0.0392	0.0196
2.6	0	0.0073	0.0111	0.0056	0.0028	0.0424	0.0212
2.7	0	0.0026	0.0148	0.0077	0.0028	0.0404	0.0202
2.8	0	0.0052	0.0148	0.0077	0.0023	0.0446	0.0223
2.9	0	0.0073	0.0148	0.0077	0.0016	0.0474	0.0237

**Table 2:** Composition of WPU dispersions.

Sample	BD, wt.%	CDPN, wt.%	DMPA, wt.%	PCL, wt.%	Sample	BD, wt.%	CDPN, wt.%	DMPA, wt.%	PCL, wt.%
1.1	0	5.0	5.0	50.8	2.1	1.2	0	5.0	50.8
1.2	0	10.0	5.0	44.9	2.2	2.3	0	5.0	44.8
1.3	0	15.0	5.0	38.9	2.3	3.3	0	5.0	39.5
1.4	0	5.0	7.5	39.3	2.4	1.2	0	7.5	39.3
1.5	0	10.0	7.5	33.3	2.5	2.3	0	7.5	33.5
1.6	0	15.0	7.5	28.0	2.6	3.3	0	7.5	28.0
1.7	0	5.0	10.0	28.6	2.7	1.2	0	10.0	28.5
1.8	0	10.0	10.0	22.7	2.8	2.3	0	10.0	22.9
1.9	0	15.0	10.0	16.8	2.9	3.3	0	10.0	16.4

**Table 3:** Vibrational band frequencies for WPU films.

Wavenumber, cm^−1^	Groups	Type of Vibration	References
1727	-C=O (PCL)	Valence vibration	[21,22]
1705	-C=O (Urethane)	Valence vibration	[23,24]
1645	-C=O (Urea)	Valence vibration	[24,25]
1635	-ONO_2_ (CDPN)	Antisymmetric valence vibrations	[26,27]
1540	-NH- (Urethane) -NH- (Urea)	Bending vibration	[23,28,29]
1275	-ONO_2_ (CDPN)	Symmetric valence vibrations	[26,27]
830	-ONO_2_ (CDPN)	Bending vibrations	[26,27]

**Table 4:** Molecular weight, hydrodynamic characteristics, and zeta potential of WPU dispersions.

Sample	BD, wt.%	CDPN, wt.%	DMPA, wt.%	PCL, wt.%	M_w_	M_w_/M_n_	Particle Size, nm	Zeta Potential, mV
1.1	0	5.0	5.0	51.2	159,000	5.4	7.3	45.1 ± 1.3
1.2	0	10.0	5.0	44.6	139,000	6.7	5.9	43.6 ± 0.6
1.3	0	15.0	5.0	39.1	128,870	9.1	3.5	42.7 ± 0.5
1.4	0	5.0	7.5	39.2	555,160	16.5	35.2	42.9 ± 0.2
1.5	0	10.0	7.5	33.5	553,760	29.5	29.9	42.9 ± 0.7
1.6	0	15.0	7.5	27.8	265,270	18.0	10.9	45.0 ± 1.1
1.7	0	5.0	10.0	28.5	257,620	18.7	78.0	42.7 ± 0.8
1.8	0	10.0	10.0	22.9	250,080	17.9	67.3	43.1 ± 0.4
1.9	0	15.0	10.0	16.8	234,180	19.2	10.0	42.5 ± 0.4
2.1	1.2	0	5.0	50.8	81,300	4.0	11.3	42.3 ± 0.1
2.2	2.3	0	5.0	44.9	85,160	4.1	8.5	43.6 ± 0.2
2.3	3.4	0	5.0	39.5	76,020	3.9	5.0	44.1 ± 0.5
2.4	1.2	0	7.5	39.2	87,350	5.1	40.8	48.3 ± 3.3
2.5	2.3	0	7.5	33.5	75,950	4.9	8.9	44.8 ± 0.8
2.6	3.4	0	7.5	27.9	74,780	4.3	4.6	45.2 ± 1.1
2.7	1.2	0	10.0	28.5	60,280	4.9	121.2	45.7 ± 3.9
2.8	2.3	0	10.0	22.7	64,280	5.2	31.9	44.5 ± 0.2
2.9	3.4	0	10.0	16.4	44,480	4.3	7.5	45.1 ± 0.3

**Table 5:** Thermophysical and thermochemical characteristics of WPU films.

Sample	BD, wt.%	CDPN, wt.%	DMPA, wt.%	PCL, wt.%	ΔH_m_, J/g	ΔH_dest_^80−265 °C^, J/g	Δm_dest_^265 °C^, %	T_g_, °C
1.1	0	5.0	5.0	51.2	0	231.9	11.4	−56
1.2	0	10.0	5.0	44.6	0	399.8	14.9	−51
1.3	0	15.0	5.0	39.1	0	474.6	20.4	−49
1.4	0	5.0	7.5	39.2	0	226.1	15.7	−56
1.5	0	10.0	7.5	33.5	0	491.1	20.9	−51
1.6	0	15.0	7.5	27.8	0	591.1	23.6	−49
1.7	0	5.0	10.0	28.5	0	244.7	18.3	−53
1.8	0	10.0	10.0	22.9	0	459.1	24.0	−55
1.9	0	15.0	10.0	16.8	0	590.3	29.0	−48
2.1	1.2	0	5.0	50.8	−19.5	0	4.5	−57
2.2	2.3	0	5.0	44.9	−28.3	0	6.8	−58
2.3	3.3	0	5.0	39.5	−29.6	0	7.2	−59
2.4	1.2	0	7.5	39.2	−27.9	0	6.3	−60
2.5	2.3	0	7.5	33.5	−32.9	0	6.5	−58
2.6	3.4	0	7.5	27.9	−22.8	0	7.7	−60
2.7	1.2	0	10.0	28.5	−49.5	0	6.9	−60
2.8	2.3	0	10.0	22.7	−34.6	0	7.2	−62
2.9	3.4	0	10.0	16.4	−50.6	0	7.0	−63

## Data Availability

The data presented in this study are available on request from the corresponding author.
